# ALK+ Anaplastic Large Cell Lymphoma of Null Cell Phenotype with Leukemic Transformation and Leukemoid Reaction

**DOI:** 10.4274/tjh.galenos.2019.2019.0021

**Published:** 2019-11-18

**Authors:** Shih-Sung Chuang, Yen-Chuan Hsieh, Hung-Chang Wu

**Affiliations:** 1 Chi-Mei Medical Centre, Department of Pathology, Tainan, Taiwan; 2 National Taiwan University Faculty of Medicine, College of Medicine, Department of Pathology, Taipei, Taiwan; 3 Taipei Medical University School of Medicine, College of Medicine, Department of Pathology, Taipei, Taiwan; 4 Chi-Mei Medical Centre, Department of Hemato-Oncology, Tainan, Taiwan

**Keywords:** ALK, Anaplastic lymphoma kinase, Anaplastic large cell lymphoma, CD30, Leukemoid reaction, Leukemic phase, Leukemic transformation

## To the Editor,

**Figure 1:**
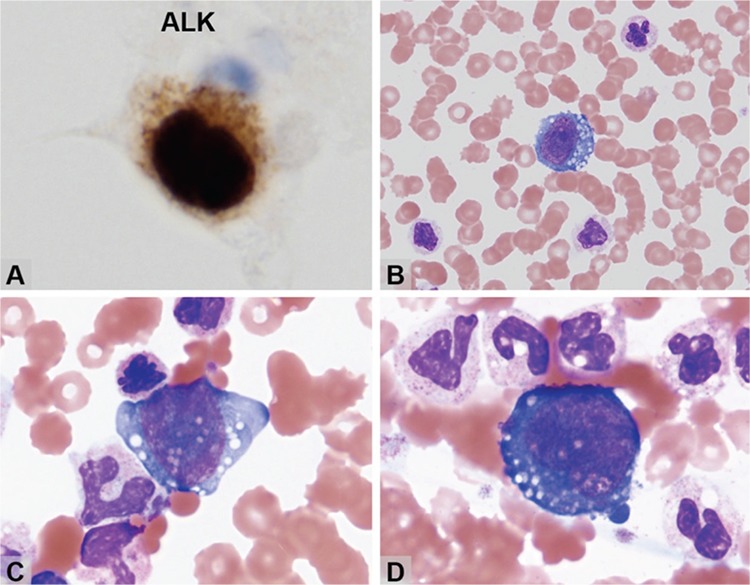
A) ALK immunostaining revealed singly scattered positive cells in addition to those in small aggregates; B-D) leukemic cells were large with vesicular nuclei, irregular nuclear contours, and vacuolated basophilic cytoplasm.

Anaplastic large cell lymphoma (ALCL) frequently involves both nodal and extranodal sites and is rarely leukemic. A 21-year-old male presented with abdominal pain. His complete blood count, which had been normal four months ago, showed increasing white cell counts from 14.9x10^9^/L to 95.5x10^9^/L in a month, with neutrophils ranging from 81.6% to 89.6%. Blood cultures were negative. Laparoscopic nodal biopsy showed sheets of medium-sized lymphocytes diffusely expressing CD30, TIA-1, granzyme B, and ALK, but not T-cell markers including CD2, CD3, CD4, CD5, CD7, CD8, and βF1, indicating ALK+ ALCL of null cell phenotype. Bone marrow biopsy showed two small aggregates of tumor cells in a background of normal tri-lineage hematopoiesis. ALK immunostaining revealed singly scattered positive cells (Figure 1A) in addition to those in small aggregates. The staining pattern was both nuclear and cytoplasmic, indicating translocation t(2;5)(p23;q35). We retrospectively reviewed the blood smear and found that 4.5% of the last peripheral smear were tumor cells, which were overlooked by the clinical laboratory. The leukemic cells were large with vesicular nuclei, irregular nuclear contours, and vacuolated basophilic cytoplasm (Figures 1B-1D). The disease progressed rapidly, and the patient passed away shortly after the first cycle of CEOP chemotherapy. In advanced diseases, ALK-positive ALCL may rarely be associated with leukemoid reaction and leukemic transformation.

